# Point-of-Care Testing in Patients with Hereditary Disorders of Primary Hemostasis: A Narrative Review

**DOI:** 10.1055/s-0044-1787976

**Published:** 2024-07-01

**Authors:** Aernoud P. Bavinck, Waander van Heerde, Saskia E.M. Schols

**Affiliations:** 1 Department of Hematology, Radboud University Medical Centre, Nijmegen, The Netherlands; 2 Department of Hematology, Radboud University Medical Centre, Hemophilia Treatment Centre Nijmegen-Eindhoven-Maastricht, Nijmegen, The Netherlands

**Keywords:** point-of-care testing, platelet function tests, blood coagulation disorders, inherited, von Willebrand disease

## Abstract

Inherited disorders of primary hemostasis, such as von Willebrand disease and congenital platelet disorders, can cause extensive, typically mucocutaneous bleeding. Assays to diagnose and monitor these disorders, such as von Willebrand factor activity assays and light transmission aggregometry, are performed in specialized hemostasis laboratories but are commonly not available in local hospitals. Due to the complexity and relative scarcity of these conventional assays, point-of-care tests (POCT) might be an attractive alternative in patients with hereditary bleeding disorders. POCTs, such as thromboelastography, are increasingly used to assess hemostasis in patients with acquired hemostatic defects, aiding clinical decision-making in critical situations, such as during surgery or childbirth. In comparison, the use of these assays in patients with hereditary hemostasis defects remains relatively unexplored. This review aims to give an overview of point-of-care hemostasis tests in patients with hereditary disorders of primary hemostasis. A summary of the literature reporting on the performance of currently available and experimental POCTs in these disorders is given, and the potential utility of the assays in various use scenarios is discussed. Altogether, the studies included in this review reveal that several POCTs are capable of identifying and monitoring severe defects in the primary hemostasis, while a POCT that can reliably detect milder defects of primary hemostasis is currently lacking. A better understanding of the strengths and limitations of POCTs in assessing hereditary defects of primary hemostasis is needed, after which these tests may become available for clinical practice, potentially targeting a large group of patients with milder defects of primary hemostasis.

Hemostasis is accomplished by a complex system, involving many interacting cells and proteins that constitute a delicate balance between hypo- and hypercoagulation. Inherited disorders of platelets or proteins involved in primary hemostasis can cause excessive, typically mucocutaneous bleeding. Von Willebrand disease (VWD) is the most common inherited bleeding disorder, with approximately 1:1000 people expressing a symptomatic bleeding tendency. 1 The prevalence of platelet function disorders (PFD) is less well known. A recent study reported an estimated prevalence of PFDs of 1:3000, based on the findings in a genome database. 2

While most patients with primary hemostasis disorders experience mild bleeding symptoms, some suffer from severe bleeding that can result in life-threatening situations in which rapid medical intervention is paramount. Making a timely and accurate diagnosis of these patients is challenging. This requires testing that is only performed in specialized clinical hemostasis laboratories.

Commonly used assays to finalize a diagnosis include light transmission aggregometry (LTA) and flow cytometry, 3 both of which are most often performed in platelet-rich plasma (PRP), necessitating blood samples to be differentially centrifuged prior to analysis, adding to the time required to perform these assays. When results are available, interpretation calls for an intricate understanding of the mechanisms underlying blood hemostasis, which is not common knowledge for most physicians. 4 As a result, misdiagnosis or significant delay between the onset of bleeding symptoms and diagnosis is common. 5

Availability of easy-to-perform assays with high specificity that rapidly inform on the potency of primary hemostasis could aid not only in the prevention of a diagnostic delay, but also in tailoring treatment in patients with an established diagnosis during a bleed or perioperatively. This would be especially useful in resource-limited countries where specialized laboratories and treatment products are scarce. 6

A wide range of assay methodologies has been employed in an effort to meet the need for such easy-to-perform assays. In some of these assays, hemostasis is assessed in whole blood under circumstances of shear stress (e.g., platelet function analyzer [PFA-100/PFA-200], total thrombus formation analysis system [T-TAS], clot-signature analyzer). Other assays, such as the Multiplate and Plateletworks, measure the reactivity of platelets and von Willebrand factor (VWF) in whole blood to specific agonists. A third category of assays evaluates global hemostasis by measuring thrombin generation or the viscoelastic properties of blood as a clot is formed, both of which are influenced by primary hemostasis. All three categories of assays are utilized as point-of-care tests (POCT) in the general population. Specific assays were found helpful to guide treatment during and after surgery 7 or labor 8 or in the care of trauma patients. 9 Additionally, POCTs of secondary hemostasis are commonly employed in monitoring anticoagulation. 10 However, due to the rarity of congenital bleeding disorders, performing sufficiently powered studies to validate such POCTs in these disorders is difficult. Furthermore, as many available POCTs are poorly standardized, aggregating results of small studies is problematic. Consequently, uncertainty regarding the validity of POCTs in hereditary bleeding disorders persists. In this review, our aim is to provide a comprehensive summary of all available POCTs used to diagnose and monitor hereditary disorders of primary hemostasis, with the main focus on VWD and PFDs.

## Methods

PubMed was searched for articles that described the use of POCTs in patients with hereditary disorders of primary hemostasis published before June 12, 2023 ( Supplementary Material , available in online version only). Articles that were available in English of any study type except narrative reviews were included.

Articles solely focusing on bleeding time without comparative analysis with other POCTs were excluded from our analysis. However, to enrich the breadth of our findings, we incorporated one review discussing the utility of bleeding time during the final drafting of the article.

### Definitions

PFD not otherwise specified was defined as a bleeding tendency in combination with abnormal aggregation in LTA but without the diagnosis of a specific platelet disorder. Mild quantitative VWD was defined as a diagnosis of VWD type 1 or “low VWF” in combination with a bleeding tendency.

### Statistical Analysis

In the calculation of overall assay sensitivity, studies were weighted according to the number of patients included. Calculations were performed with Microsoft Excel.

**Fig. 1:**
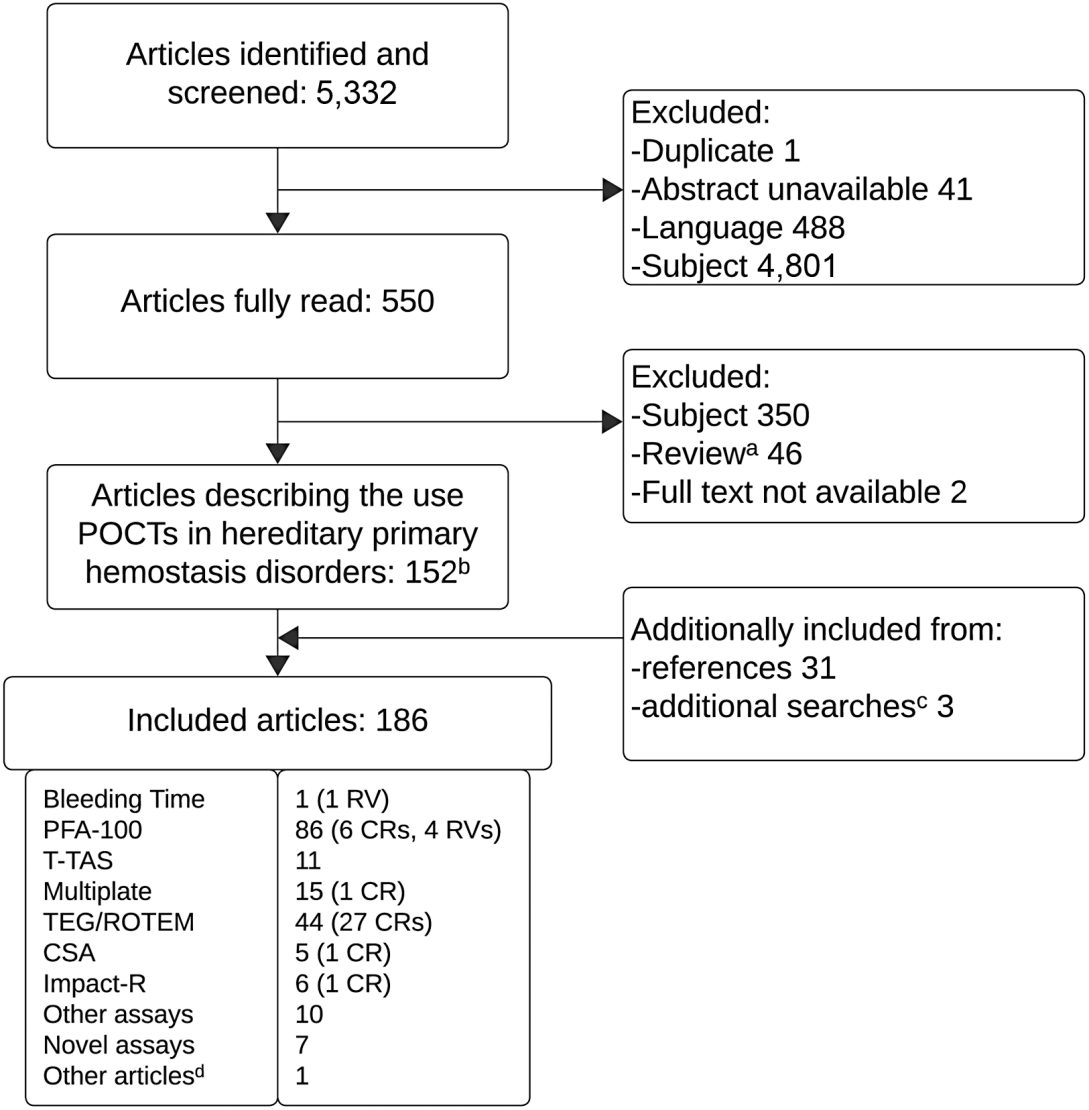
Overview of search and included articles. “Language” indicates non-English articles. “Subject” indicates not related to topic. CR, case report; CSA, clot signature analyzer; PFA, platelet function analyzer; POCT, point-of-care test; ROTEM, rotational thromboelastometry; RV, review; TEG, thromboelastography; T-TAS, total Thrombus formation analysis system. ^a^ indicates two reviews with meta-analysis aspects were included. 79 96 ^b^ indicates one article was included despite the full text being unavailable, as the main outcomes were clear from the abstract. 11 ^c^ indicates one and two additional articles were identified through targeted searches on PubMed for “Platelet mapping” 12 and “whole blood lumi,*” 13 14 respectively. ^d^ indicates a questionnaire study on what assays are used in practice for patients with (suspected) inherited platelet disorders. 3

Graphs were created using R version 4.1.3. Fig. 1 was created using Lucidchart. Other figures were created with Adobe Illustrator 2023.

## Results

After an extensive literature research, 186 articles were included in this review. A flowchart of the articles included in this review is presented in Fig. 1 .

Various POCTs to assess primary hemostasis have been employed over the years. Bleeding time, first described approximately 3,000 years ago by Huang Ti, 11 has long been considered a fundamental tool in this regard. In this test, hemostasis is evaluated by creating a small cut in the skin of the patient and monitoring the time it takes until bleeding stops. Many variations of the bleeding time have been developed over the years, with later methods trying to automate and standardize the technique, to reduce variability in test results. Among these revised methods, the Ivy and Template bleeding time are notable for their extensive use in the past century. Nevertheless, patient discomfort and interoperator variability remained persistent problems in all these techniques. Rodgers and Levin stated in their extensive review in 1990 that there are no clear criteria for the use of bleeding time, as there was no evidence that it could accurately predict bleeding and aid in monitoring the effect of treatment. 12

**Fig. 2:**
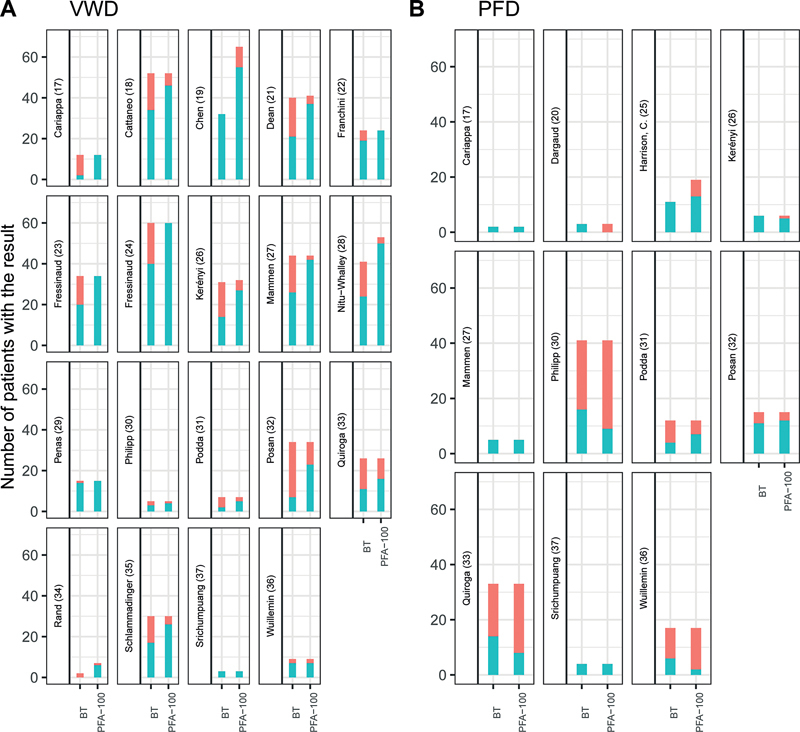
( **A** and **B** ) Overview of studies directly comparing platelet function analyzer (PFA) and bleeding time (BT) in patients with von Willebrand disease ( **A** ) and platelet function disorders (PFDs) ( **B** ). Bars represent number of patients tested with the assay. Patients with an abnormal result (true-positive) are depicted in blue, patients with a normal result (false-negative) are depicted in red.

**Fig. 3:**
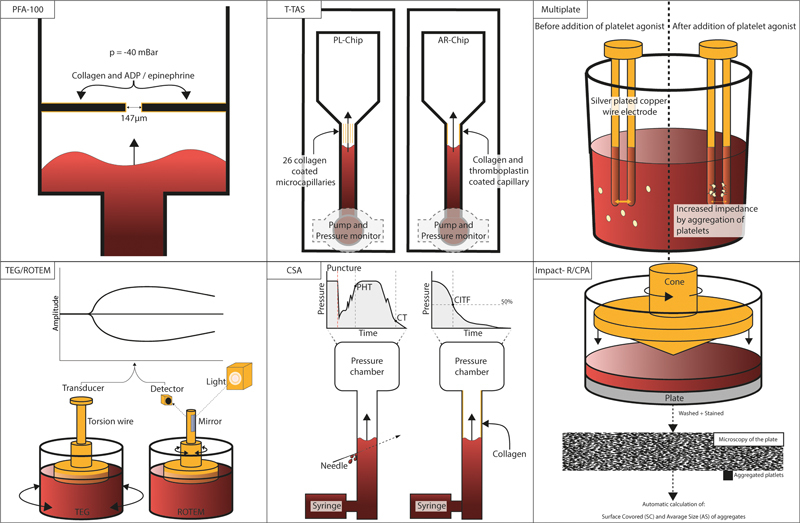
Schematic overview of the described POC assays. CIFT, collagen-induced thrombus formation time; CPA, cone and plate(let)/impact-R analyzer; CSA, clot signature analyzer; CT, clotting time; PHT, platelet hemostasis time; PFA, platelet function analyzer; POC, point-of-care; ROTEM, rotational thromboelastometry; TEG, thromboelastography.

Several laboratory methods have been described in an attempt to mimic bleeding time in vitro. For example, in the capillary thrombometer whole blood was pumped back and forth through a glass column until clot formation resulted in occlusion of the tube. 13 14 Similarly, whole blood was passed through a woven polyethylene terephthalate 15 or glass fiber 16 filter to measure “filter bleeding time.” These methods showed promising results to detect severe primary hemostasis disorders but did not find widespread use. Nowadays, bleeding time has mostly been replaced by the PFA-100 and PFA-200. This device is reported to be more sensitive in patients with VWD compared with bleeding time. However, for other primary hemostasis defects, diagnostic performance is comparable to bleeding time ( Fig. 2 ). 17 18 19 20 21 22 23 24 25 26 27 28 29 30 31 32 33 34 35 36 37 Other devices that hold resemblance to the bleeding time, such as the T-TAS, clot signature analyzer (CSA), cone and plate(let)/impact-R analyzer (CPA), and experimental microfluidic flow chambers, 38 have been used specifically in patients with hereditary primary hemostasis disorders. Multiplate and viscoelastic assays employ different methods to assess hemostasis and have also been studied in these patients. In this review, the mechanism of action of these POCTs is first described ( Fig. 3 and Table 1 ). Next, articles describing the results of their use in hereditary disorders of primary hemostasis will be discussed.

**Table 1:** Overview of point-of-care tests included in this review

Platform	Assay	Activating agents	Sample used (volume) a	Other reagents	Shear rate	Reaction time b	Outcomes	Studied in
PFA-100/PFA-200 (Siemens Healthineers, Germany)	Epinephrine cartridge	Collagen and epinephrine	Citrated WB (800 µL)	None	5000/s	<8 min	Closure time	VWD; GT; BSS; SPD; δ-SPD/HPS; PSD; (Mild) PFD NOS; gray platelet syndrome, aspirin-like defect, others ( Supplementary Table S2 , available in online version only)
	ADP cartridge	Collagen and ADP						
	P2Y12 cartridge	ADP and PGE1						
Total thrombus-formation analysis system, T-TAS (Zacros Fujimori Kogyo Co, Japan)	PL Chip	Collagen	BAPA-incubated WB (320 µL)	None	1500/s	<10 min	Occlusion start time (OST); occlusion time (OT); area under the curve (AUC)	VWD; GT; BSS; δ-SPD; Mild PFD NOS
	AR Chip	Collagen and tissue thromboplastin	Recalcified WB (480 µL)	CTI	600/s	<30 min		
	HD chip	Collagen and tissue thromboplastin		DNDS	1200/s	<30 min		
Multiplate analyzer (Roche Diagnostics, Switzerland)	ADPtest	ADP	Hirudin or heparin-treated WB (300 µL)	None	NR	<10 min	Area under aggregation curve; Velocity (AU/min); aggregation units (AU)	VWD; GT; BSS; PSD (Mild) PFD NOS
	TRAPtest	TRAP						
	RISTOtest	Ristocetin						
	ASPItest	AA						
	COLtest	AA and collagen						
TEG (Haemonetics Corporation, USA)	Kaolin TEG	Kaolin	Recalcified WB (360 µL)	None	0.1/s	Real time	R-time; K-time; α-angle; tMRTG; MRTG; MA; CL30, CL60 ( Fig. 4 )	VWD; GT; BSS; PSD, SPD; Wiskott-Aldrich syndrome
	Tissue factor TEG	Tissue factor		(CTI)				
	Rapid TEG	Tissue factor and Kaolin		None				
	Native TEG	None						
	Kaolin TEG with heparinase	Kaolin		Heparinase				
	Functional fibrinogen TEG	Kaolin		Abciximab				
	Platelet Mapping	Either 1) Kaolin 2) Reptilase, FXIII (ADP or AA)	Recalcified WB (360 µL) and heparinized WB (720–1,080 µL)	None			% Inhibition	VWD, GT, BSS, Grey platelet syndrome
ROTEM (Pentapharm GmbH, Germany)	EXTEM	Tissue factor	Recalcified WB (300 µL)	(CTI)	0.1/s	Real time	Clotting time (CT); clot formation time (CFT); α-angle; t-MaxVel; MaxVel; Max clot firmness (MCF); Ly30, Ly60 ( Fig. 4 )	VWD; GT; BSS; PSD; May–Hegglin anomaly
	INTEM	Kaolin/ellagic acid + phospholipids		None				
	NATEM	None						
	FIBTEM	Tissue factor		Cytochalasin-D				
	HEPTEM	Kaolin/ellagic acid + phospholipids		Heparinase				
	APTEM	Tissue factor		Aprotinin				
ROTEM platelet module (TEM innovations, Germany)	ARATEM	AA	Whole blood with citrate, heparin or hirudin (150 µL)		NR	6 min	Area under the aggregation curve; Maximum slope (MS); A6 (Ohm)	GT	
	ADPTEM	ADP							
	TRAPEM	TRAP					
Clot signature analyzer (CSA) (Xylum Corporation, USA)	Collagen channel	Collagen	WB (3ml)	None	Variable, >1500/s	<30 min	Collagen-induced thrombus formation time (CITF time)	VWD; GT; SPD; HPS
	Puncture channel	Mechanical puncture of channel			variable, >10,000/s		CT; Platelet hemostasis time	
Impact-R Cone and Plate(let) analyzer (Matis Medical Inc., Belgium)	NA	None	Citrated WB (130 µL)	None	1,800/s	NR	Surface area covered (SC); Area size (AS)	VWD; GT; SPD; (Mild) PFD NOS

Abbreviations: δ-SPD, δ storage pool disease; AA, arachidonic acid; ADP, adenosine diphosphate; AR, atheroma; BAPA, benzylsulfonyl-d-arg-pro-4-amidinobenzylamide, inhibits FX and thrombin; BSS, Bernard–Soulier syndrome; CTI, corn-derived trypsin inhibitor, inhibits FXII; DNDS, 4,4'-Dinitrostilbene-2,2'-disulfonic acid disodium salt, inhibits erythrocyte precipitation; GT, Glanzmann thrombasthenia; HPS, Hermansky–Pudlak syndrome; NA, not applicable; NR, not reported; P2Y12, PFD NOS, platelet function disorder not otherwise specified; PGE1, prostaglandin E1; PL, platelet; PSD, platelet secretion disease; TRAP, thrombin receptor-activating peptide; VWD, von Willebrand disease; VWF, von Willebrand factor; WB, whole blood.

a Volume needed per assay performed.

b Excluding the time to prepare the platform for the assay (e.g., preheating).

**Fig. 4:**
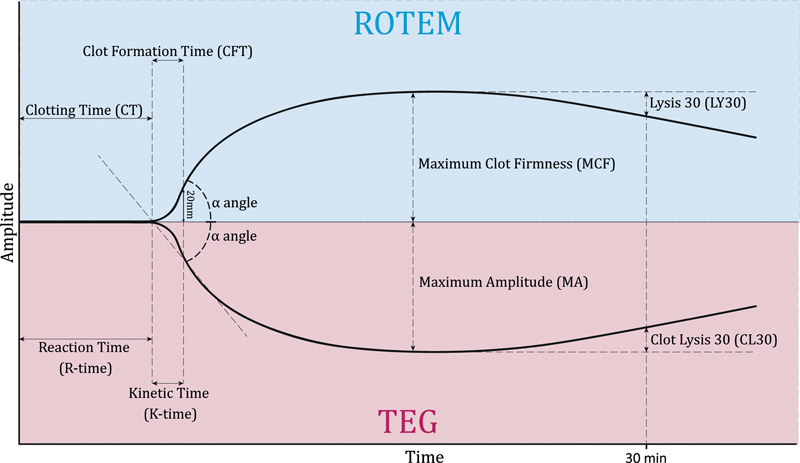
Example of a thromboelastography (TEG) and rotational thromboelastometry (ROTEM) tracing. All parameters reflect on distinct aspects of coagulation. Initiation phase: reaction-time (R-time) and clotting time (CT), the time from start of the assay until an amplitude of 2 mm is reached. Propagation phase: kinetic-time (K-time) and clot formation time (CFT), time from 2-mm amplitude until 20-mm amplitude; a-angle, slope between R-time/CT and K-time/CFT; clot strength, maximal amplitude (MA) and maximal clot firmness (MCF), highest amplitude reached; fibrinolysis, clot lysis 30 (CL30) and lysis 30 (LY30), decrease of amplitude after 30 minutes compared with MA/MCF.

Interestingly, several available point-of-care (POC) hemostasis assays have never been studied in patients with congenital primary hemostasis defects: the Global Thrombosis Test, Hemodyne analyzer, Plateletworks, Ultegra Rapid Platelet Function Assay and its follow-up the Verifynow, ReoRox, Sonoclot, and the Quantra Hemostasis Analyzer. Some of these tests utilize methods that were able to identify patients with inherited primary bleeding disorders. For instance, in Plateletworks, platelet count measured before and after addition of agonists determines the platelet activation to these agonists. While this method holds promise in patients with hereditary platelet disorders, 39 Plateletworks has never been validated in these patients.

## Mechanism of Action of the Point-of-Care Tests

An overview of characteristics of the assay techniques is depicted in Table 1 . A schematic representation of the mechanism of the assays is shown in Fig. 3 .

### Platelet Function Analyzer (PFA-100, PFA-200)

The PFA-100 system was first described in 1995 as a follow-up of the Thrombostat-4000, 40 building upon a technique to assess primary hemostasis as described by Kratzer and Born in 1985. 41 Citrated whole blood is inserted into one of two different cartridges and aspirated through a 150-µm aperture in a collagen and either epinephrine (EPI) or adenosine diphosphate (ADP)-coated membrane. This procedure generates a shear force of 5,000 to 6,000/seconds, presumably activating VWF. The closure time, the time until the aperture is closed, is a marker of the potency of primary hemostasis. 40 An updated version of the PFA-100, the PFA-200, was released mid-2010s. While the user interface has been changed significantly, test results were shown to be similar to the older model. 42 43 Furthermore, an additional cartridge designed to test P2Y12 function has been created (INNOVANCE PFA P2Y). Development of this cartridge was incentivized by the need for a convenient assay to measure the effect of P2Y12-inhibiting drugs.

### Total Thrombus Formation Analysis System

This device was introduced in 2011 as a method to assess thrombus formation under high and low shear force. Two distinct chips are used: the PL-chip with a collagen-coated flow-chamber, aimed to assess platelet function in whole blood under conditions of high shear force. The second chip called the AR-chip is coated with tissue thromboplastin, with the main goal to assess factor-based coagulation in whole blood containing corn trypsin inhibitor. In both chips, whole blood is perfused through a channel, while the pressure within this channel is continuously monitored. The time to reach predefined pressure levels serves as a surrogate for the onset of thrombus formation and subsequent channel occlusion. These parameters constitute the main outcomes of the assay.

### Multiplate

A technique called multiple electrode aggregometry is used. This assay was first described in 2004 in an abstract on the 10 ^th^ Erfurt Conference on Platelets by Calatzis et al 44 as an improvement of the method that was described by Cardinal and Flower in 1979. 45 The reactivity of platelets in whole blood to different agonists is assessed by continuously measuring the electrical impedance in the test cell following addition of these agonists. As platelets adhere to two pairs of electrodes, the electrical impedance increases, thereby reflecting the platelet responsiveness to the added agonist.

### Viscoelastic Tests

Testing of the viscoelastic properties of blood over time informs on entire coagulation process from initiation to fibrinolysis. Hartert described this technology as early as in 1948. 46 Technologic advancement has rekindled interest in this technique at the end of the 20 ^th^ century. Two viscoelastic techniques have been studied extensively in patients with hereditary bleeding disorders: thromboelastography (TEG) and rotational thromboelastometry (ROTEM). In both TEG and ROTEM, plasma or citrated whole blood is placed in a heated cuvette in which a pin is suspended. In TEG, the cuvette rotates, whereas in ROTEM, the pin rotates. As coagulation occurs and fibrin networks are formed, the viscoelasticity of the sample increases. In TEG, the increasing viscoelasticity leads to a higher conveyance of the rotating cuvette with blood to the pin. This, in turn, causes the pin to track the movements of the cuvette more closely. An electromagnetic transducer monitors the movement of the pin. In contrast, in ROTEM the movement of the rotating pin is increasingly impeded as the viscoelasticity of the blood rises, resulting in diminished rotation of the pin. The detection is achieved optically in a commonly used ROTEM platform (ROTEM delta). For both TEG and ROTEM, the viscoelasticity of the sample over time is depicted in characteristic curves, from which several parameters can be identified. The most commonly used parameters and their definitions are outlined in Fig. 4 . Depending on the agent added to initiate coagulation, various assays are recognized ( Table 1 ).

Modifications of TEG and ROTEM have been developed to specifically assess platelet function: TEG Platelet Mapping and ROTEM Platelet Analysis. In TEG Platelet Mapping, the maximal amplitude (MA) obtained after addition of different agonists and inhibitors of hemostasis is compared. While this method involves relatively many steps for a POCT, it has shown promise in a POC setting for trauma patients 47 and cardiac surgery patients. 48 Firstly, a standard kaolin TEG is performed to determine the maximal clot strength after activation of platelets by contact pathway-mediated generation of thrombin (MA _thrombin_ ). MA in this assay represents the clot strength that can be attained after maximal platelet stimulation. Secondly, the clot strength due to fibrin network formation without platelet activation is determined (MA _fibrin_ ). This is accomplished by adding reptilase and FXIII to heparinized blood. Reptilase converts fibrinogen to fibrin in a thrombin-independent manner, whereas heparin prevents the activation of thrombocytes by thrombin. This last assay is repeated in the presence of either arachidonic acid (AA) or ADP, to assess the clot strength that is reached after stimulation of platelets by these agents (MA _AA_ and MA _ADP_ ). The results of the assays are compared to determine platelet aggregation after stimulation with AA or ADP as a percentage of maximal platelet aggregation (Equation 1), or the percentage of AA or ADP receptors inhibited (100% − percentage of aggregation).



The ROTEM platelet module takes a different approach and measures impedance aggregometry in response to AA, ADP, and thrombin receptor-activating peptide simultaneously with conventional ROTEM analysis.

### Clot Signature Analyzer

The pressure over time in two oil-filled pressure chambers connected to distinct channels perfused with whole blood is examined. 49 One of these channels is coated with collagen. Flow of blood through the channels causes pressure to build up in the pressure chambers. Consequently, as the channels occlude due to thrombus formation, the pressure in the pressure chambers decreases to zero. The time until the pressure drops to 50% in the collagen channel (collagen-induced thrombus formation time) and to 10% in the uncoated channel (clotting time—CT) are two of the main outcomes of the assay. A unique feature of this assay is that the channel not coated with collagen is punctured with a needle to mimic vascular injury. As a result, the flow to the associated pressure chamber declines, resulting in a temporary decrease in pressure, until blood coagulation restores the continuity of the channel. The time from puncturing the channel until recovery of pressure is called the platelet hemostasis time and constitutes the third main outcome of this assay. In fact, this “tube bleeding time” was used to successfully detect Glanzmann thrombasthenia (GT) prior to release of the CSA. 50

### Impact-R/Cone and Plate(let) Analyzer

Blood is placed in a polystyrene well and shear force is applied with a circulating Teflon cone. After 2 minutes, the well is washed and stained. The amount of adhered and aggregated platelets as determined by surface coverage and the average size of adhered particles are calculated by a computer.

In the next section, an overview of studies reporting on the use of these assays in patients with hereditary disorders of primary hemostasis is presented.

## Results of the Point-of-Care Tests in Patients with Hereditary Disorders of Primary Hemostasis

### Platelet Function Analyzer (PFA-100, PFA-200)

Apart from being influenced by aberrations in primary hemostasis, this assay's main outcome is reported to be influenced by several physiological parameters, such as platelet and hematocrit level, 32 43 51 blood group, 52 time during the day, 36 age (neonates having shorter closure time), 53 and physical exercise. 54 Currently, PFA-100 is one of the most used POC hemostasis assays in patients with suspected hereditary disorders of primary hemostasis. 55 However, a Worldwide Survey of ISTH members found that only 58% of all responding hemostasis laboratories were using the PFA-100. 3

**Fig. 5:**
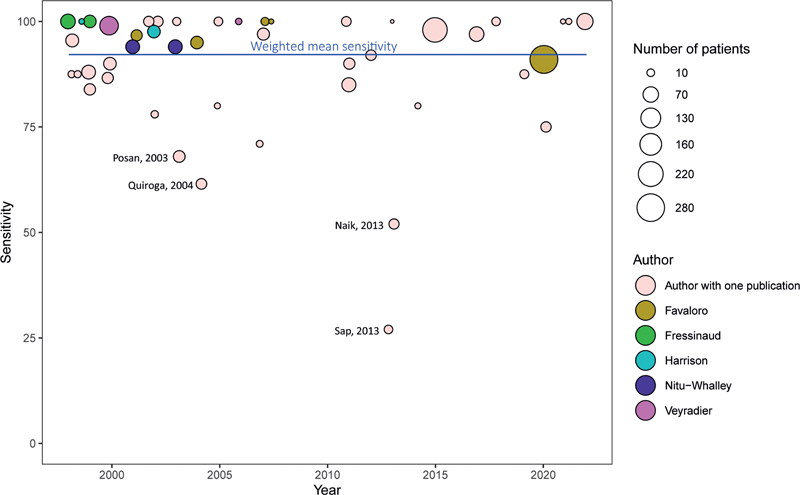
Reported sensitivity of platelet function analyzer in von Willebrand disease patients. Only studies that performed both the epinephrine and adenosine diphosphate cartridge were included unless sensitivity was 100%, despite only one of these cartridges being used, as in these cases using both cartridges would not have altered the sensitivity. Studies written by authors who published multiple publications on this subject are depicted in colors identifying the particular author. Size of the points signifies the number of patients included in the study. Author and year of publication are depicted for studies reporting a sensitivity < 70%.

PFA-100 is extensively studied in patients with (suspected) VWD. 17 18 19 21 22 23 24 26 27 28 29 30 31 32 33 34 35 36 37 43 51 52 53 56 57 58 59 60 61 62 63 64 65 66 67 68 69 70 71 72 73 74 75 76 77 78 Favaloro et al reported an overall sensitivity of 83.2% and 91.5% of the ADP and EPI cartridge respectively in a review in 2008. 79 A current analysis including all articles published before and after 2008 yielded similar test performance outcomes, as shown in Fig. 5 and Supplementary Table S1 (available in online version only). Two recent large retrospective studies incorporating the data of over 10 years of experience confirmed the high diagnostic value of PFA-100 in detecting VWD. 43 52 Normal PFA-100 results are especially rare in patients with abnormal VWF function or a lower quantity of large molecular VWF multimers. 24 26 32 35 60 78 80 81 In cases of mild type 1 VWD the false-negative rate is higher. Sap et al reported a sensitivity of only 29%, specifically in this group of patients. 77 However, all patients diagnosed with VWD type 1 in this study had (near)-normal VWF:Ag levels, whereas VWF:RCo levels were markedly decreased. Accordingly, the patients' VWF profile was more in line with type 2 VWD, raising questions about the precise VWD diagnoses of patients in this study. Only patients with VWD type 2N consistently exhibited normal test results, 24 26 67 unless VWF levels were also low. This observation aligns with expectations, since PFA is not able to detect secondary hemostasis defects, such as FVIII deficiency, which is the main outcome of type 2N VWD. 24 53 56 80 As the PFA primarily functions as a screening tool, it does not contribute to identifying the specific subtype of VWD in any given patient. 28 Nor can the PFA-100 distinguish VWD from PFDs. However, comparison of PFA-100 results before and after administration of DDAVP has been used to help differentiate patients with severe type 1 and classical type 2 VWD. 81

**Fig. 6:**
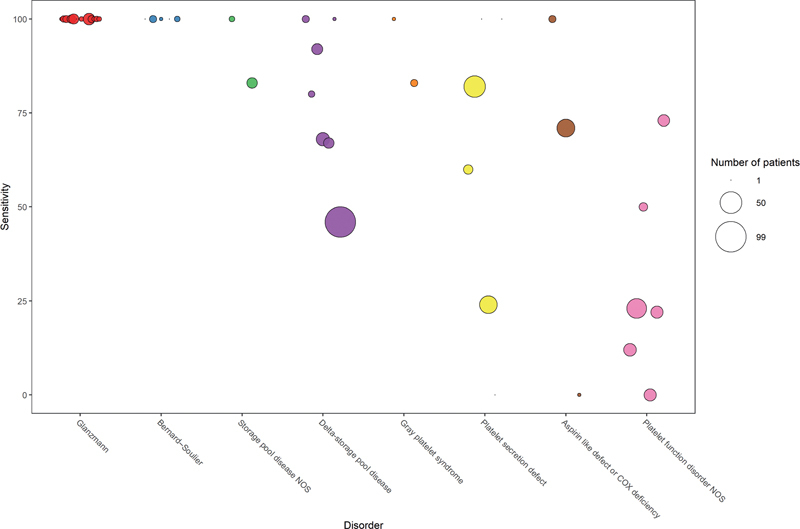
Reported sensitivity of the platelet function analyzer in patients with platelet function disorders. Either an abnormal test result with the epinephrine or adenosine diphosphate cartridge is considered a positive test. Size of the points represents number of patients included in the study. Colors represent the disease studied. NOS, not otherwise specified.

The diagnostic utility of the PFA-100 has been studied in a variety of diseases other than VWD. An overview of the published literature on test results in primary hemostasis disorders other than VWD is presented in Fig. 6 and Supplementary Table S2 (available in online version only). Karger et al found a pooled sensitivity of 82.5% and 66.9% of EPI and ADP cartridge, respectively, for detecting primary hemostasis defects. 82 Individuals diagnosed with severe PFDs, such as GT 17 24 26 27 31 37 51 55 57 60 66 68 75 83 84 85 86 and Bernard–Soulier syndrome (BSS), 51 55 60 75 87 consistently exhibited prolonged closure time with both cartridges. In fact, blood of GT patients was not able to occlude the PFA-100's aperture even after transfusion of normal pooled platelets. 85 It was suggested that the patients' GPIIbIIIa-deficient platelets compete with the transfused platelets for adhesion to the membrane. However, once adhered, the patients' platelets lack the capacity to aggregate effectively, resulting in the inability to form a stable blood clot and obstruct the PFA's aperture.

In patients with milder bleeding disorders, such as platelet secretion disorders or storage pool disorders (SPD), diagnosis can be missed when solely relying on PFA-100. 17 20 24 25 26 30 31 32 33 36 51 55 57 60 65 66 68 84 88 89 90 91 92 93 94 95 On the whole, the EPI cartridge demonstrates greater sensitivity than the ADP cartridge in detecting primary hemostasis defects. In practice, isolated prolongation of the closure time in the EPI cartridge is often attributed to drug-induced COX-1 inhibition such as that caused by aspirin. This pattern is also frequently observed in patients with mild VWD or PFDs. Using both cartridges simultaneously offers the benefit of providing information on the severity of the underlying disorder. If only the EPI cartridge shows abnormal results, a diagnosis of severe VWD or PFD is unlikely.

The added value of the INNOVANCE P2Y cartridge in patients with hereditary disorders of primary hemostasis is uncertain. While it showed improved sensitivity for moderate–severe P2Y12 defects in a small study, 94 the detection rate of PFDs and VWD did not increase by adding this cartridge to the diagnostic protocol. 66

The PFA-100 has also been used to detect primary hemostasis disorders prior to surgery. In a meta-analysis on preoperative screening for bleeding disorders in pediatric patients, PFA-100 performed best of the screening methods studied, which also included several questionnaires, bleeding time, and activated partial thromboplastin time. 96 However, the authors stated that given the general lack of high-quality studies, care should be taken to draw firm conclusions. The largest included study in this meta-analysis found low diagnostic value of PFA in screening unselected preoperative patients and argued it had led to unnecessary delay of surgical procedures. 97 While this study reported false-positive results to be problematic, others found insufficient sensitivity to be the largest problem in preoperative screening. 84 Conversely, Koscielny et al reported the PFA-100 to be useful in screening before surgery: they were able to reduce perioperative blood product use by utilizing PFA-100 in a protocol to detect hemostasis defects in unselected patients scheduled for surgery and to monitor treatment prior to surgery. 98

Apart from identifying patients with VWD or PFDs, PFA-100 has been used to monitor treatment in patients with hereditary bleeding disorders. The extent of normalization of closure time correlates with the increase in VWF activity (including VWF:RCo) and VWF:Ag in patients with VWD type 1 treated with DDAVP. 18 22 23 34 66 80 99 100 101 In patients with type 2 VWD, however, closure time remained prolonged after DDAVP in the subset of patients with normalization of VWF:Ag, possibly due to a persistent lack of high molecular VWF multimers. 80 81 99 102 Monitoring treatment with plasma-derived VWF (pd-VWF) concentrate with PFA-100 is unreliable, as most concentrates do not contain high molecular weight VWF multimers. 18 23 71 99 103 In contrast, a few recent studies suggested that monitoring treatment with recombinant VWF concentrate is promising, as this product does contain high molecular weight VWF multimers and can lead to correction of prolonged PFA CTs. 104 105

While most studies assessed the validity of PFA-100 in monitoring treatment compared with conventional assays, there is limited literature on the correlation with clinical outcomes. Some studies reported that PFA-100 results correlated with the severity of bleeding tendency. 31 64 106 This finding was not, however, confirmed by others. 25 107 108 109 The correlation between the change in closure time after treatment with clinical outcomes is even less clear. Weston et al reported that DDAVP might prevent bleeding even in patients with unchanged closure time. 110 Similarly, Hanebutt et al noted that all three patients in whom closure time did not shorten after DDAVP administration did not experience subsequent bleeding complications. 100 Accordingly, questions surrounding the validity of PFA-100 to monitor treatment remain.

Some patients with clinically elevated bleeding tendency have abnormal PFA-100 results, while test results in all other hemostasis studies are normal. Heubel-Moenen et al showed significant abnormalities in extensive multiparameter microfluidic assays in a subset of these patients. 111 In some cases, the test results exhibited abnormalities to a comparable extent as observed in patients with GT. The authors hypothesized that these patients have multifactorial defects in shear-dependent mechanisms of primary hemostasis, which cannot be detected with traditional static hemostasis assays.

Taken altogether, the suboptimal sensitivity in patients with mild PFDs hampers the utility of PFA-100 in assessing patients with suspected hereditary bleeding disorders and has compelled several authors and the ISTH Platelet Physiology Subcommittee to advise against using PFA-100 in screening for hereditary bleeding disorders. 30 33 59 112 113 We, however, concur with the conclusions of Favaloro et al who recently stated that the main purpose of the PFA-100/200 is to rapidly exclude VWD. 42

### Total Thrombus Formation Analysis System

Ogiwara et al were the first to describe the use of this device in patients with hereditary bleeding disorders. 114 They showed that all five tested patients with various types of VWD had impaired thrombus formation in the PL-chip. Of interest, thrombus formation was also abnormal in one patient diagnosed with VWD type 2N. However, as the VWF:RCo of this patient was 32 IU/dL, the cause of this result might have been low VWF activity in addition to low FVIII activity levels. Later studies confirmed high sensitivity of T-TAS in patients with VWD, especially in patients with deficiency of high molecular weight multimers. 115 116 Additionally, a correlation between bleeding tendency and T-TAS outcomes was found in patients with VWD type 1. 117 However, sensitivity in those with mild VWD type 1 and (VWF: Ag > 25%) was limited. 115 As such, it was questioned whether T-TAS has added value over PFA-100 in patients with VWD. Charpy et al performed the only study that directly compared both assays. 68 While this small study reported a relatively low sensitivity of PFA-100 (75%), the sensitivity of PL-chip was even lower (42%). An explanation for the low sensitivities might be that all patients with VWF:GP1bR < 50% were considered to have VWD in this study. As such, many included patients did not exhibit a bleeding tendency; the median ISTH Bleeding Assessment Tool score in patients with VWF:GP1bR level of 30 to 39% was just one.

T-TAS has two notable advantages over PFA-100 in patients with VWD. Nakajima et al reported that the AR-chip of the T-TAS provides the possibility to identify patients with VWD type 2N and assess their bleeding risk. 118 Furthermore, the AR-chip showed normalization of test outcomes after infusion of pd-VWD/FVIII concentrate, in contrast to the PFA-100. 119 T-TAS might therefore be more suitable to monitor treatment with pd-VWD/FVIII concentrate.

Superiority of T-TAS over PFA-100 appears more clearly in patients with mild PFDs, although only a few relevant studies are available. Similarly to PFA-100, results of T-TAS were abnormal in all patients with GT or BSS. 116 In addition, the diagnostic performance of T-TAS was better in mild thrombocytopathies compared with PFA-100. T-TAS test results were abnormal in 80 to 100% of patients with δ-SPD 120 121 and in 70 to 82% of patients with abnormalities in (lumi-)LTA. 68 122 Nonetheless, a sensitivity of 70% still limits the general utility of this assay as a screening tool.

### Multiplate

In VWD, using ristocetin as an activating agent, diagnostic performance of Multiplate in most studies was excellent in all patients, except in those patients with a mild subtype 1. 64 123 124 Similar to PFA-100 and T-TAS, sensitivity was notably lower in patients with mild type 1 VWD. 64 65 However, Valarche et al reported normal Multiplate results even in patients with VWD type 2A and 2M. 76 Notably, four out of five patients with normal results in this study had a VWF activity of ≥40%, which could potentially account for the observed low sensitivity. An advantage of multiplate compared with other POCTs is the ability to identify patients with VWD type 2B, in whom increased aggregation to low-dose ristocetin was seen. 64 76 However, a study by Nakajima et al raised doubts about the technique's reliability, as none of the three tested patients with VWD type 2B demonstrated increased aggregation in response to low-dose ristocetin. 116

The ability of Multiplate to simultaneously measure the response to different agonists is especially interesting. In theory, it could allow differentiation of various platelet disorders, whereas other POCTs only measure general platelet function. Multiplate consistently showed abnormal results in patients with GT and BSS, 84 86 125 126 demonstrating high agreement with results obtained by LTA. However, limited sensitivity in patients with mild PFDs was seen. Only 40% of patients with abnormalities in LTA were identified with Multiplate in one study. 84 In another study, only 20% of pediatric patients with mild bleeding disorders had abnormal Multiplate results. 65 In this last study, the addition of Multiplate to a protocol for screening in patients with a suspected bleeding disorder did not increase sensitivity of the screening protocol and was therefore deemed ineffective. Lastly, Al Ghaithi et al reported that Multiplate only identified 3 out of 20 patients with PFDs detected by lumi-LTA. 127 This outcome is not surprising, as the Multiplate much alike conventional LTA does not measure ATP release.

Insufficient sensitivity in mild platelet disorders seems to restrict the usability of this device in patients with (suspected) hereditary bleeding disorders. Interestingly, some limitations might have been resolved in a different system, the Chrono-log Whole Blood Lumi-Aggregometer (Chrono-log Corporation), as this platform does allow for performing concurrent whole blood impedance aggregometry and ATP release measurements. Using this system, reduced ATP secretion was found in a patient with Hermansky–Pudlak syndrome (HPS—a genetic syndrome characterized by albinism and absence of platelet delta granules). 128 Further reports on the use of the Chrono-log Whole Blood Lumi-Aggregometer in patients with hereditary bleeding disorders are limited to studies describing its use in the screening of patients with heavy menstrual bleeding. 129 130 Conclusions, however, cannot be drawn from these studies regarding the diagnostic performance of the device, as conventional lumi-aggregometry in plasma was not performed in these studies.

### Viscoelastic Assays (Thromboelastography and Rotational Thromboelastometry)

The lack of high shear stress in TEG and ROTEM is thought to hamper their utility in assessing primary hemostasis disorders, especially in VWD. Nonetheless, several studies have described the use of these techniques in these disorders, with varying success.

In VWD, prolonged coagulation initiation (R-time or CT) and decreased propagation (K-time and CFT) have been observed. 131 132 133 However, the test parameters were found to correlate with FVIII activity levels but not with VWF activity levels 132 and the tracings normalized in VWD type 3 patients after suppletion with FVIII concentrates 131 ; these abnormalities are therefore likely explained at least in part by FVIII deficiency and not by the alterations in VWF-mediated hemostasis.

Interestingly, while overall it is thought that TEG and ROTEM produce similar, although not interchangeable results, studies using ROTEM reported lower sensitivity in patients with VWD compared with TEG. Strikingly, even some patients with VWD type 2 or 3 exhibited normal results with ROTEM. 132 134 135 This difference in performance was clearly seen in a study involving 100 VWD patients, whose hemostasis was tested with both TEG and ROTEM. 132

Relatively many studies described the use of TEG and ROTEM in patients with PFDs. However, the vast majority of these studies consists of case reports involving patients undergoing surgical procedures 136 137 138 139 140 141 142 143 144 145 or during peripartum care. 146 147 148 149 150 151 152

In GT, viscoelastic assays unequivocally have shown decreased clot strength (MA/maximal clot firmness). 126 153 154 155 156 Using TEG Platelet Mapping, it was even possible to identify a GT patient with a mild bleeding phenotype from four other patients with more severe bleeding tendencies, 83 which suggests that this technique might be an attractive method to monitor GT patients. Utility of viscoelastic assays to monitor treatment in GT is further supported by the observation that maximal clot strength improved after treatment with platelet transfusions, 157 158 fibrinogen, 154 155 and recombinant factor XIII. 154 Addition of recombinant factor VIIa (rFVIIa) did not normalize clot strength despite adequate clinical outcome in one study, which suggests limited utility of viscoelastic assays altogether in monitoring rFVIIa treatment in GT. 159 This last study is an exception on the general lack of studies correlating the changes in test results after treatment with clinical outcomes, despite anecdotal evidence suggesting that hemostasis might still be impaired in spite of normalization of test results. 143

In BSS, clot strength is mostly unaltered, but the coagulation propagation phase is affected (increased K-time/CFT and decreased α angle). 126 153 Only one study reported the effects of treatment on viscoelastic assays in BSS: rFVIIa improved the initiation and propagation phase, whereas fibrinogen improved maximal clot strength in addition to increasing the propagation. 150

In contrast to the results of the assays in these severe platelet disorders, blood of patients with mild bleeding disorders often produces normal tracings. As such, the added value of these assays was found to be limited in screening for mild bleeding disorders. 135

Hypothetically, addition of TEG Platelet Mapping and the ROTEM platelet module could improve the diagnostic value of TEG and ROTEM. However, their use in patients with hereditary disorders is exclusively reported in small (case) studies, 126 143 145 147 153 obstructing the formulation of definite conclusions regarding their utility.

### Discontinued Assays

The CSA and Impact-R (CPA) have previously been used in patients with hereditary bleeding disorders. However, these systems are not commercially available nowadays. Nonetheless, the lessons learned with these systems might prove valuable in developing novel testing platforms.

#### Clot Signature Analyzer

The CSA was successfully used to monitor two patients with HPS during labor. However, a subsequent larger study reported insufficient sensitivity of the assay in patients with HPS. 160 The latest study on CSA in patients with hereditary bleeding disorders was performed by Fricke et al in 2004, who studied the diagnostic accuracy of CSA in a large group of patients with various hemostasis defects. 161 Sensitivity was high in patients with VWD (91%) and coagulation factor deficiencies (92%), while it was lower in patients with various PFDs (69%). Regarding VWD patients, only patients with type 1 VWD had false-negative results. Specificity was acceptable, with 89% of healthy controls showing normal results. Since 2004 there have been no additional publications using CSA in patients with hereditary bleeding disorders.

#### Impact-R (Cone and Plate(let) Analyzer)

Platelet deposition was found to be highly dependent on surface immobilization of plasma VWF on the polystyrene plate 162 and proved to be useful in detecting VWD, GT, and afibrinogenemia. 163 In a trial involving pediatric patients with diverse primary hemostasis defects, the assay demonstrated a notable capacity to reliably rule out bleeding disorders, with a sensitivity of 90%. However, the specificity was comparatively lower at 67.5%. 164 A modification of the assay was later proposed, performing the Cone and Plate(let) assay both before and after the addition of platelet agonists to the sample. 165 This modification might allow for differentiation of different PFDs, but further studies confirming its validity in patients with PFDs are lacking.

### Novel Assays

A full overview of new POC techniques to monitor coagulation is outside the scope of this review. However, some novel assays have shown promising results specifically in patients with hereditary bleeding disorders and will be discussed briefly.

In several novel devices, hemostasis was monitored by perfusing whole blood through microfluidic channels. Some experimental flow chamber devices were tested in patients with VWD or suspected primary hemostasis disorders. 38 166 167 168 In these microfluidic devices, whole blood was perfused through capillaries with varying shear stress, owing to the specific geometry of the capillaries. Video microscopy was used to monitor the area of the capillaries covered by a growing thrombus, reflecting hemostatic potency. These assays showed high sensitivity even in patients with VWD type 1, signifying potential added value over currently available POCTs. In the device of Grabowski et al, blood is incubated with anti-GPIIb and ALEXA 555-conjuncted anti-mouse antibodies. Using epifluorescence this device was able to monitor total volume of thrombus formation in addition to monitoring the area covered. Twenty-four patients with low VWF were evaluated with this assay. None of the patients with normal platelet adhesion in this device developed clinical bleeding, whereas 7/16 patients with low platelet adhesion did. Therefore, this assay might have a role in predicting bleeding tendency in patients with VWD or low VWF, although its utility as a POCT is limited by the need for incubation prior to testing. 169

Another device that optically measures clot formation in flow chambers was called the clotMAT. 170 In this device, micropillars are placed inside the channels. As blood perfuses the channels, clots are formed between these micropillars. In addition to optically monitoring the clot formation, this device also measured the contractile force of the clot and the clot stiffness. Early experiments with plasma of type 2A VWD patients showed that these additional measurements could provide more insight in the hemostatic status of these patients. However, further validation is needed.

## General Discussion and Study Limitations

In this narrative review of available POCTs, aggregated data result in a remarkable lack of an outstanding POCT that is able to detect mild hereditary disorders of primary hemostasis, whereas sensitivity of all assays was excellent in severe disorders, such as GT, BSS, and severe VWD. Unfortunately, most patients referred for an increased bleeding tendency in developed countries will harbor mild hemostatic defects, and therefore, we believe that currently available POCTs are of limited value in screening patients with suspected bleeding disorders.

Several factors are likely to contribute to the suboptimal performance of the POCTs. An ideal POCT should assess hemostasis under (near-)physiological circumstances, incorporating all mechanical forces, 171 proteins, and cells that contribute to hemostasis in vivo. However, deviations from this ideal scenario are apparent across all discussed POCTs.

First, the effects of endothelial cells and the subendothelial matrix are not incorporated in any of the assays. Although in PFA, T-TAS and CSA platelets are exposed to collagen to resemble in vivo platelet adhesion, these methodologies do not comprehensively address the hemostatic functions attributed to the endothelium. 172

In addition, Multiplate and viscoelastic assays are conducted under static conditions. The crucial role of high shear rate in unfolding VWF and subsequent platelet adhesion is thus neglected in these assays, 173 with the outcome that these assays show low sensitivity in mild quantitative VWF disorders. Interestingly, even in the assays that harbor higher shear rates such as PFA-100 and T-TAS, mild VWD is often missed. It should be noted that in all assays the shear rates applied were still low when compared with the shear rates observed in vascular injuries, thus deviating from in vivo conditions. 174

Lastly, the use of citrated blood samples in most POCTs is a cause for concern. Beyond its role in inhibiting thrombin generation, the nonphysiological low ionized calcium environment induced by citrate significantly impairs platelet function. Immediately after blood draw, platelet reactivity was lower in citrated whole blood 175 and citrated PRP 176 compared with samples anticoagulated with other agents. Relatively small increases in citrate concentration further blunted platelet reactivity in LTA. 177 Paradoxically, reactivity to the weak platelet agonist ADP can be increased by using citrated blood, 178 due to augmented thromboxane A _2_ production 179 and decreased ectonucleotidase activity. 180 While this effect might be beneficial in the detection of mild PFDs, it constitutes an artifact that deviates from the physiological in vivo situation. Additionally, there are concerns regarding the stability of citrated samples. Platelet function begins to deteriorate rapidly in citrated PRP as early as 2 hours after blood drawing, while it remains relatively preserved in PRP anticoagulated with heparin, 176 hirudin, or benzylsulfonyl-d-Arg-Pro-4-amidinobenzylamide (BAPA, a dual inhibitor of FXa and thrombin). 181 Platelet function shows similar deterioration in citrated whole blood samples and samples treated with hirudin, 182 while the samples treated with heparin 176 or BAPA 183 have shown greater stability. BAPA-treated samples even showed stable PFA results for up to 24 hours after blood draw. 183

Given these limitations of the current generation of POCTs and the unsatisfactory results in patients with mild hemostatic defects, negative assay results do not reliably exclude an underlying disorder, and positive results require follow-up testing to make a definite diagnosis. Therefore, the ability of POCTs to significantly affect clinical decision-making is limited. However, we do not exclude a potential use of these assays in appropriate settings, as the findings of this review suggest the capability of these POCTs in monitoring primary hemostasis perioperatively. Also, some devices, such as the PFA, may be useful for quick exclusion of VWD. Most importantly, it is crucial that any assay result in medicine is considered in conjunction with the clinical data of the individual patient. As such, while not being able to perfectly rule out any disorder, these POCTs might still aid clinicians in diagnostic decision-making and in personalizing treatment.

Several studies assessed the influence of prohemostatic treatment on POCT results and suggested utility of these assays in monitoring. However, it might be premature to conclude that such monitoring strategies are beneficial, as trials correlating the results of POCTs after treatment with meaningful clinical outcomes are lacking. Alternatively, POCTs might provide valuable information in emergency situations when specialized hemostasis testing is unavailable. To this end, TEG and ROTEM are nowadays frequently used to steer treatment of patients with acquired coagulopathy in the emergency department or operating theatre. While numerous case reports suggest utility of POCTs in patients with hereditary bleeding disorders of primary hemostasis during surgery, no clinical prospective trials have been performed on this subject.

We acknowledge that definitive conclusions are difficult to draw from the presented data due to several limitations in methodology of the available studies and results. Firstly, due to the rare nature of many of the inherited disorders of primary hemostasis, most of the included studies only described a limited number of patients. We opted to include these articles to provide preliminary evidence and offer directions for future research. However, the importance of exercising care in drawing conclusions based on these small-scale studies should not be understated. Moreover, aggregation of the small studies to provide more substantial evidence is complicated due to the high variability in the methodology and definitions used in these studies. For instance, patients with a bleeding tendency, VWF:Ag 30 to 50%, with normal VWF activity/Ag ratios, are classified as VWD type 1 patients by the current ASH ISTH NHF WFH (American Society of Hematology, International Society on Thrombosis and Haemostasis, National Hemophilia Foundation, World Federation of Hemophilia) 2021 guidelines. 184 Historically, these patients were considered to have “low VWF” and were not diagnosed with VWD. To allow for summation of the available literature, we decided to combine all patients with mild quantitative VWF defects in one group to calculate overall assay performance. Nevertheless, this approach comes at the cost of reduced homogeneity within this group. Furthermore, while relatively many studies described the use of viscoelastic assays, aggregation of these studies is complicated, due to considerable variability in the assay protocols.

Secondly, we calculated the overall assay performance of the PFA-100 in an explorative fashion and did not perform a true systematic meta-analysis. As such, we weighted studies solely based on number of patients included and did not consider study quality in the calculation of the overall sensitivity.

Thirdly, several researchers were involved in multiple studies included in this review. This poses a risk that patients are included multiple times.

Lastly, we provided a detailed description of studies that used POCTs in patients with hereditary disorders of primary hemostasis but did not incorporate the vast amount of literature on these assays in other disorders. Extrapolation of results of this additional literature might have aided in substantiating conclusions and identifying additional uses of POCTs, such as differentiating between different types of bleeding disorders.

## Conclusion

Overall, POC testing for primary hemostasis is a dynamic field and specific problems of the current generation of devices might be solved by future improvements and inventions. In recent years, discoveries in microfluidics 185 have paved the way for a new generation of “Lab on a Chip” devices to assess hemostasis. 186 These advancements have enabled the development of platforms that could potentially revolutionize the care for patients with hereditary platelet disorders, such as a disk-shaped device that automatically generates PRP from inserted whole blood and subsequently performs LTA. 187 Additionally, future assays performed with whole blood in microchannels coated with endothelial cells under high shear forces could provide the means for more realistic in vitro hemostasis testing. 188 It remains to be seen how effectively these innovative devices will translate from the laboratory setting to real-world clinical applications, ultimately filling the yet unmet need for a truly reliable and easy-to-use primary hemostasis assay.
