# Supplementary material for: Point-of-Care Testing in Patients with Hereditary Disorders of Primary Hemostasis: A Narrative Review
Source: Semin Thromb Hemost. 2024 Jul 1;51(5):541–59. doi: 10.1055/s-0044-1787976 (PMC12165736; doi:10.1055/s-0044-1787976)
Supplement: Supplementary file 1 — Supplementary Material [file 10-1055-s-0044-1787976-s03242.pdf]

## Supplementary Appendix

### Search Strategy

The initial PubMed search was performed with the following terms: (((“Blood Coagulation Disorders, Inherited”[Mesh] OR “Blood Coagulation Disorders”[Mesh] OR “coagulation dis*”[tiab] OR “bleeding dis*”[tiab] OR “factor deficienc*”[tiab] OR “factor dysfunction”[tiab] OR “thrombopathy”[tiab] OR “hemophilia”[tiab] OR “haemophilia”[tiab]) OR (“Blood Coagulation Tests”[Mesh] OR “coagulation tes*”[tiab] OR “aptt”[tiab] OR “PT”[tiab] OR “prothrombin time”[tiab] OR “activated partial thromboplastin time”[tiab] OR “INR”[tiab] OR “thrombin generation”[tiab] OR “TEG”[tiab] OR “ROTEM”[tiab] OR “TGA”[tiab] OR “thromboelastography”[tiab] OR “PFA”[tiab] OR “platelet function analy*”[tiab] OR “fibrinogen”[tiab] OR “aggregometry”[tiab] OR “factor VIII”[tiab] OR “factor 8”[tiab] OR “factor IX”[tiab] OR “factor 9”[tiab] OR “ACT”[tiab] OR “activated clotting time”[tiab])) AND (“Point-of-Care Testing”[Mesh] OR “POCT”[tiab] OR “POC”[tiab] OR “point-of-care”[tiab] OR “point of care”[tiab] OR “near patient”[tiab] OR “near-patient”[tiab] OR “bedside”[tiab]))

We found that many relevant articles did not include the terms “point-of-care,” “near patient,” “bedside,” or similar terms in the title, abstract, or MESH terms. Therefore, we expanded the search to allow for articles that did not state these terms to be found: (((“Platelet Function Tests”[Mesh] OR “T-TAS”[tiab] OR “PFA-100”[tiab] OR “PFA-200”[tiab] OR “multiplate”[tiab]) OR (“Thrombelastography”[Mesh] OR “thromboelastogra*”[tiab] OR “thromboelastome*”[tiab] OR “TEG”[tiab] OR “ROTEM”[tiab])) AND (“Blood Coagulation Disorders, Inherited”[Mesh] OR “coagulation dis*”[tiab] OR “bleeding dis*” OR “hemophilia” OR “haemophilia” OR “thrombopathy” OR “Glanzmann” OR “Bernard-Soulier” OR “Bernard Soulier” OR “Willebrand Disease” OR “VWD” OR “factor V def*” OR “FV def*” OR “factor VII def*” OR “FVII def*” OR “FX* def*” OR “factor X* def*” OR “fibrinogen dis*” OR “prothrombin def*” OR “PAI-1 def*” OR “storage pool dis*” OR “gray platelet syndrom*”)) OR ((((“Blood Coagulation Disorders, Inherited”[Mesh] OR “Blood Coagulation Disorders”[Mesh] OR “coagulation dis*”[tiab] OR “bleeding dis*”[tiab] OR “factor deficienc*”[tiab] OR “factor dysfunction”[tiab] OR “thrombopathy”[tiab] OR “hemophilia”[tiab] OR “haemophilia”[tiab]) OR (“Blood Coagulation Tests”[Mesh] OR “coagulation tes*”[tiab] OR “aptt”[tiab] OR “PT”[tiab] OR “prothrombin time”[tiab] OR “activated partial thromboplastin time”[tiab] OR “INR”[tiab] OR “thrombin generation”[tiab] OR “TEG”[tiab] OR “ROTEM”[tiab] OR “TGA”[tiab] OR “thromboelastography”[tiab] OR “PFA”[tiab] OR “platelet function analy*”[tiab] OR “fibrinogen”[tiab] OR “aggregometry”[tiab] OR “factor VIII”[tiab] OR “factor 8”[tiab] OR “factor IX”[tiab] OR “factor 9”[tiab] OR “ACT”[tiab] OR “activated clotting time”[tiab])) AND (“Point-of-Care Testing”[Mesh] OR “POCT”[tiab] OR “POC”[tiab] OR “point-of-care”[tiab] OR “point of care”[tiab] OR “near patient”[tiab] OR “near-patient”[tiab] OR “bedside”[tiab])))

Additional searches on PubMed for “Verifynow,” “Global thrombosis test,” “Plateletworks,” “Rapid Platelet Function Assay,” “ReoRox,” “Hemodyne,” “Clot signature analysis,” and “Quantra” did not yield additional relevant results.

 © 2024. Thieme. All rights reserved.

**Supplementary Table S1** Sensitivity of platelet function analyzer-100/200 in von Willebrand disease

| Author | *N* | Sensitivity (%) | | | Specificity (%) | | | Subtype (*N* tested positive/*N* tested) | | | | | | | | |
|---|---|---|---|---|---|---|---|---|---|---|---|---|---|---|---|---|
| | | EPI | ADP | Combined | EPI | ADP | Combined | Mild quantitative defect[a] | Type 2: subtype unknown | Type 2A | Type 2B | Type 2N[b] | Type 2M | Type 3 | Acquired | Platelet type |
| Carcao 1998[53] | 8 | 88 | 88 | 88 | – | – | – | 6/7 | – | – | – | – | – | 1/1 | – | – |
| Fressinaud 1998[24 c] | 61 | 97 | 100 | 100 | 96 | 99 | – | 36/36 | – | 10/10 | 3/3 | 0/2 | – | 4/4 | 5/5 | 3/3 |
| Mammen 1998[27] | 44 | 96 | NR | 96 | 89 | – | – | – | – | – | – | – | – | – | – | – |
| Rand 1998[34] | 8 | 88 | 88 | 88 | – | – | – | 6/7 | – | – | – | – | – | 1/1 | – | – |
| Cattaneo 1999[18] | 52 | 87 | 88 | 88[d] | – | – | – | 17/22 | – | 6/6 | 9/9 | – | 6/6 | 6/6 | – | – |
| Favaloro 1999[56 c] | 9 | 100 | 100 | 100 | 62 | 84 | – | 3/3 | – | 1/1 | 4/4 | – | | 1/1 | – | – |
| Fressinaud 1999[23 c] | 41 | 100 | 100 | 100 | – | – | – | 24/24 | – | 4/4 | 3/3 | – | 6/6 | 4/4 | – | – |
| Harrison 1999[51 c] | 4 | 100 | 100 | 100 | 100 | 100 | – | – | – | – | – | – | – | 3/3 | – | – |
| Kerényi 1999[26] | 31 | 84 | 73 | 84[d] | – | – | – | 19/22 | – | 2/2 | 2/4 | 0/1 | – | 3/3 | – | – |
| Dean 2000[21] | 41 | 88 | 90 | 90 | – | – | 95 | 20/24 | 13/13 | – | – | – | – | 4/4 | – | – |
| Schlammadinger 2000[35] | 30 | 87 | 66 | 87 | 97 | 100 | – | 19/22 | – | 2/2 | 2/3 | – | – | 3/3 | – | – |
| Veyradier 2000[67 c] | 110[e] | 94[e] | 99[e] | 99[e] | 96 | 98 | – | NR/56 | NR/3 | NR/17 | NR/9 | 0/2 | NR/8 | NR/6 | NR/9 | – |
| Favaloro 2001[80 c] | 30 | 97 | 77 | 97[d] | 69 | 91 | – | 17/18 | – | 3/3 | 7/7 | | | 1/1 | – | 1/1 |
| Nitu-Whalley 2001[61 c] | 52 | NR | NR | 94 | – | – | – | NR/32 | – | NR/6 | NR/1 | | NR/12 | NR/1 | – | – |
| Buyukasik 2002[57] | 25 | 100 | 100 | 100 | 85 | – | – | – | – | – | – | – | – | – | – | – |
| Franchini 2002[22] | 24 | 100 | 100 | 100 | – | – | – | 24/24 | – | – | – | – | – | – | – | – |
| Harrison 2002[60 c] | 41 | NR | NR | 98 | 80 | 82 | – | 32/33 | – | – | 2/2 | – | – | 6/6 | – | – |
| Wuillemin 2002[36] | 9 | 78 | 56 | 78[d] | 88 | 100 | – | 5/7 | – | – | – | – | – | 2/2 | – | – |
| Cariappa 2003[17] | 12 | 100 | 80 | 100 | 97 | 80 | – | – | – | – | – | – | – | – | – | – |

(*Continued*)

 © 2024. Thieme. All rights reserved.

**Supplementary Table S1** (*Continued*)

| Author | *N* | Sensitivity (%) | | | Specificity (%) | | | Subtype (*N* tested positive/*N* tested) | | | | | | | | |
|---|---|---|---|---|---|---|---|---|---|---|---|---|---|---|---|---|
| | | EPI | ADP | Combined | EPI | ADP | Combined | Mild quantitative defect[a] | Type 2: subtype unknown | Type 2A | Type 2B | Type 2N[b] | Type 2M | Type 3 | Acquired | Platelet type |
| Nitu-Whalley 2003[28 c] | 53 | 94 | 94 | 94 | – | – | – | NR/32 | – | NR/7 | NR/1 | | NR/12 | NR/1 | – | – |
| Posan 2003[32] | 34 | 65 | 61 | 68 | 89 | 90 | – | 12/22 | 6/7 | – | – | – | – | 2/2 | 3/3 | – |
| Favaloro 2004[69 c] | 41 | 80 | 95 | 95 | – | – | – | 16/18 | 19/19 | – | – | – | – | 3/3 | – | 1/1 |
| Quiroga 2004[33] | 26 | 50 | 62 | 62 | – | – | 98 | 16/26 | – | – | – | – | – | – | – | – |
| Penas 2005[29] | 15 | 100 | 100 | 100 | – | – | – | – | – | – | – | – | 15/15 | – | – | – |
| Philip 2005[30] | 5 | NR | NR | 80 | – | – | 89 | – | – | – | – | – | – | – | – | – |
| Veyradier 2006[62 c] | 6 | 100 | 100 | 100 | – | – | – | 6/6 | – | – | – | – | – | – | – | – |
| Favaloro 2007[71 c] | 12 | 100 | 100 | 100 | – | – | – | 5/5 | – | – | 2/2 | | 1/1 | 4/4 | – | – |
| Favaloro 2007[70 c] | 3 | 100 | 100 | 100 | – | – | – | – | – | – | – | – | – | – | – | 3/3 |
| Perel 2007[73] | 37 | 97 | NR | 97 | – | – | – | 35/35 | – | 1/1 | – | – | 0/1 | – | – | – |
| Podda 2007[31] | 7 | 71 | 71 | 71 | – | – | – | NR/5 | – | NR/2 | – | – | – | – | – | – |
| Akin 2011[72 f] | 20 | 100 | 100 | 100 | – | – | – | 20/20[g] | – | – | – | – | – | – | – | – |
| Chen 2011[19] | 55 | 85 | 67 | 85[d] | – | – | – | – | – | – | – | – | – | – | – | – |
| Valarche 2011[76 h] | 30 | 76 | 90 | 90 | – | – | – | 11/12 | 9/11 | – | 3/3 | – | – | – | 1/1 | 3/3 |
| Koessler 2012[66] | 25 | 80 | 76 | 92 | – | – | – | NR/21 | NR/1 | – | – | – | NR/1 | NR/2 | – | – |
| Kılıç 2013[75] | 2 | 100 | 50 | 100 | – | – | – | 2/2 | – | – | – | – | – | – | – | – |
| Naik 2013[59] | 23 | 30 | 52 | 52 | – | – | 89 | NR/23 | – | – | – | – | – | – | – | – |
| Sap 2013[77] | 14 | 29 | 21 | 29[d] | – | – | 100 | 4/14 | – | – | – | – | – | – | – | – |
| Gursel 2014[63] | 5 | 80 | 40 | 80 | – | – | 98 | 4/5 | – | – | – | – | – | – | – | – |

 © 2024. Thieme. All rights reserved.

**Supplementary Table S1** (*Continued*)

| Author | *N* | Sensitivity (%) | | | Specificity (%) | | | Subtype (*N* tested positive/*N* tested) | | | | | | | | |
|---|---|---|---|---|---|---|---|---|---|---|---|---|---|---|---|---|
| | | EPI | ADP | Combined | EPI | ADP | Combined | Mild quantitative defect[a] | Type 2: subtype unknown | Type 2A | Type 2B | Type 2N[b] | Type 2M | Type 3 | Acquired | Platelet type |
| Ardillon 2015[52] | 213 | 91 | 96 | 98 | 54 | 53 | 40 | 106/119[i] | – | 115/117[i] | 75/77[i] | – | 125/128[i] | 7/7[i] | – | – |
| Nummi 2017[64] | 61 | 93 | 97 | 97 | – | – | – | 15/17 | 4/4 | 16/16 | 8/8 | 1/1 | 3/3 | 13/13 | – | – |
| Casonato 2018[58] | 14 | 100 | NR | 100 | – | – | – | – | – | – | – | – | 14/14 | – | – | – |
| Haas 2019[65] | 16 | 88 | 19 | 88[d] | – | – | – | 15/16 | – | – | – | – | – | – | – | – |
| Charpy 2020[68] | 24 | 75 | NP | 75[d] | 88 | – | – | – | – | – | – | – | – | – | – | – |
| Favaloro 2020[43] [c] | 285 | 88 | 75 | 91 | – | – | 69 | 163/188 | | 21/21 | 15/15 | | 17/17 | 11/11 | 27/27 | 6/6 |
| Bakr 2021[74] | 5 | 100 | 60 | 80 | – | – | | – | – | – | – | – | – | – | – | – |
| Srichumpuang 2021[37] | 3 | 100 | 100 | 100 | – | – | 96 | – | – | – | – | – | – | – | – | – |
| Geevar 2022[78] | 79 | NR | NR | 100 | – | – | – | NR[j] | NR[j] | – | – | – | – | 62/62 | – | – |
| Patients tested | 1745 | – | – | – | – | – | – | 635 | 54 | 66 | 63 | 6 | 63 | 134 | 36 | 17 |
| Positive result (%) | – | – | – | – | – | – | – | 552 (87) | 51 (94) | 66 (100) | 60 (95) | 1 (17) | 62 (98) | 134 (100) | 36 (100) | 17 (100) |
| Weighted average sensitivity (%) | – | 88 | 84 | 92 | – | – | – | – | – | – | – | – | – | – | – | – |

Abbreviations: ADP, adenosine diphosphate cartridge; EPI, epinephrine cartridge; NP, not performed; NR, not reported; VWD, von Willebrand disease.
[a]Both patients with VWD subtype 1 or "low VWF" in combination with a bleeding phenotype are included in this category.
[b]Patients with subgroup 2N were not included in the overall sensitivity calculation and the total number of patients per study.
[c]The inclusion of multiple articles authored by the same individual in this table raises concerns about the potential duplication of test results.
[d]Sensitivity obtained if both cartridges were combined is not reported, highest sensitivity of either EPI or ADP cartridge was used as approximation of combined sensitivity.
[e]Results include two patients with subtype 2N and three with subtype 2N or 3.
[f]Study in VWD subtype three carriers.
[g]5/20 patients were diagnosed as having severe subtype 1 or subtype 2.
[h]Only 17/30 patients tested with the EPI cartridge. Only considering patients with both tests resulted in combined sensitivity of 82% (14/17).
[i]The same patients were tested multiple times, test results in subtypes are not included in "Patients tested" and "Positive result (%)".
[j]Not reported how many patients with type 1 or 2 VWD were included in PFA analysis; however, all of these patients had prolonged closure time.

 © 2024. Thieme. All rights reserved.

**Supplementary Table S2** Sensitivity of platelet function analyzer-100/200 in platelet function disorders

| Author | Year | Patients | Disorder | Cartridges | Sensitivity | | | Specificity | | |
|---|---|---|---|---|---|---|---|---|---|---|
| | | *N* | | | EPI cartridge | ADP cartridge | Combined | EPI cartridge | ADP cartridge | Combined |
| Weighted mean | | 77 | Glanzmann thrombasthenia (GT) | | 100% | 98% | 100% | NA | NA | NA |
| Fressinaud[24] | 1998 | 2 | GT | EPI; ADP | 100% | 100% | 100% | 95% | 95% | – |
| Mammen[27] | 1998 | 5 | GT | EPI; ADP | 100% | 100% | 100% | – | – | 88.3% |
| Harrison[51 a] | 1999 | 6 | GT | EPI; ADP | 100% | 100% | 100% | 100% | 100% | – |
| Kerényi[26] | 1999 | 1 | GT | EPI; ADP | 100% | 100% | 100% | – | – | – |
| Harrison[60 a] | 2002 | 8 | GT | EPI; ADP | 100% | 88% | 100% | 80% | 82% | – |
| Buyukasik[57] | 2002 | 11 | GT | EPI; ADP | 100% | 100% | 100% | 85% | | – |
| Cariappa[17] | 2003 | 1 | GT | EPI; ADP | 100% | 100% | 100% | 97% | 80% | – |
| Podda[31] | 2007 | 1 | GT | EPI; ADP | 100% | 100% | 100% | – | – | – |
| Callaghan[83] | 2008 | 3 | GT | EPI; ADP | 100% | 100% | 100%[b] | – | – | – |
| Koessler[66] | 2012 | 1 | GT | EPI; ADP; P2Y[c] | 100% | 100% | 100%[b] | – | – | – |
| Kılıç[75] | 2013 | 1 | GT | EPI; ADP | 100% | 100% | 100%[b] | – | – | – |
| Albanyan[86] | 2015 | 15 | GT | NR | – | – | 100%[b] | – | – | – |
| Perez Botero[55] | 2017 | 6 | GT | EPI; ADP | – | – | 100% | – | – | – |
| Moenen[84] | 2017 | 3 | GT | EPI; ADP | – | – | 100% | – | – | – |
| Al-Battat[85] | 2018 | 4 | GT | EPI; ADP | 100% | 100% | 100% | – | – | – |
| Charpy[68] | 2020 | 6 | GT | EPI | 100% | | | 94% | – | – |
| Srichumpuang[37] | 2021 | 3 | GT | EPI; ADP | 100% | 100% | 100% | – | – | 96% |
| Weighted mean | | 14 | Bernard–Soulier syndrome (BSS) | | 90% | 100% | 100% | NA | NA | NA |
| Harrison[51 a] | 1999 | 1 | BSS | EPI; ADP | 100% | 100% | 100% | 100% | 100% | – |
| Harrison[60 a] | 2002 | 6 | BSS | EPI; ADP | 100% | 100% | 100% | 80% | 82% | – |
| Cakı Kılıç[75] | 2013 | 2 | BSS | EPI; ADP | 50% | 100% | 100% | – | – | – |
| Bragadottir[87] | 2015 | 1 | BSS | EPI; ADP | 100% | 100% | 100% | – | – | – |
| Perez Botero[55] | 2017 | 4 | BSS | EPI; ADP | | | 100% | – | – | – |
| *Liang*[189] | *2007* | *21* | *Heterozygous loss of GP Ib-V-IX (due to velocardiofacial syndrome)* | *EPI; ADP* | *62%* | *57%* | *71%* | – | – | – |
| Weighted mean | | 16 | Storage pool disease (SPD) | | 87% | 62% | 87% | NA | NA | NA |
| Fressinaud[24] | 1998 | 4 | SPD | EPI; ADP | 100% | 100% | 100% | | | |
| Harrison[60 a] | 2002 | 12 | SPD | EPI; ADP | 83% | 50% | 83%[b] | 80% | 82% | |
| Weighted mean | | 156 | Hermansky–Pudlak syndrome (HPS)/delta-storage pool disease (δ-SPD) | | 51% | 26% | 58% | NA | NA | NA |

 © 2024. Thieme. All rights reserved.

**Supplementary Table S2** (*Continued*)

| Author | Year | Patients | Disorder | Cartridges | Sensitivity | | | Specificity | | |
|---|---|---|---|---|---|---|---|---|---|---|
| | | *N* | | | EPI cartridge | ADP cartridge | Combined | EPI cartridge | ADP cartridge | Combined |
| Harrison[51 a] | 1999 | 6 | HPS | EPI; ADP | 100% | 86% | 100% | 100% | 100% | – |
| Kerényi[26] | 1999 | 5 | HPS | EPI; ADP | 80% | 0% | 80% | | | – |
| Harrison[60 a] | 2002 | 13 | HPS | EPI; ADP | 92% | 54% | 92% | 80% | 82% | – |
| Harrison[25] | 2002 | 19 | HPS | EPI; ADP | – | – | 68% | – | – | – |
| Perez Botero[55] | 2017 | 12 | HPS/ δ-SPD | EPI; ADP | – | – | 67% | – | – | – |
| Acharya[88] | 2008 | 2 | δ-SPD | EPI; ADP | – | – | 100%[d] | – | – | 95% |
| Sladky[89] | 2012 | 99 | δ-SPD | EPI; ADP | 41% | 20% | 46% | – | – | |
| Weighted mean | | 8 | Gray platelet syndrome | | 100% | 100% | 87% | NA | NA | NA |
| Harrison[60] | 2002 | 2 | Grey platelet syndrome | EPI; ADP | 100% | 100% | 100% | 80% | 82% | – |
| Perez Botero[55] | 2017 | 6 | Grey platelet syndrome | EPI; ADP | – | – | 83% | – | – | – |
| Weighted mean | | 95 | Platelet secretion defect (PSD) | | 56% | 35% | 51% | NA | NA | NA |
| Harrison[60] | 2002 | 10 | PSD | EPI; ADP | 50% | 60% | 60%[e] | 80% | 82% | – |
| Buyukasik[57] | 2002 | 49 | PSD | EPI; ADP | 82% | 36% | 82%[e] | 85% | – | – |
| Cariappa[17] | 2003 | 1 | PSD associated with Jacobsen's syndrome | EPI; ADP | 100% | 100% | 100% | 97% | 80% | – |
| Quiroga[33] | 2004 | 33 | PSD/aggregation defect | EPI; ADP | 18% | 24% | 24% | – | – | – |
| Santos[90] | 2011 | 1 | PSD | NR | – | – | 0% | – | – | – |
| Perez Botero[55] | 2017 | 1 | PSD associated with Jacobsen's syndrome | EPI; ADP | – | – | 100% | – | – | – |
| *Podda[31]* | *2007* | *11* | *PSD $n = 8$, SPD $n = 3$* | *EPI; ADP* | *55%* | *0%* | *55%* | – | – | – |
| Weighted mean | | 42 | Aspirin-like defect | | 70% | 37% | 72% | NA | NA | NA |
| Fressinaud[24] | 1998 | 6 | Aspirin-like defect | EPI; ADP | 100% | 50% | 100% | 95% | 95% | |
| Rolf[91] | 2009 | 34 | Aspirin-like defect | EPI; ADP | 65% | 35% | 71% | – | – | 77% |
| Knöfler[92] | 2014 | 2 | Aspirin-like defect | NR | | | 0% | – | – | |
| Weighted mean | | 133 | Platelet function disorder (PFD) not otherwise specified (NOS)[f] | | 33% | 10% | 27% | NA | NA | NA |
| Wuillemin[36] | 2002 | 17 | PFD NOS | EPI; ADP | 12% | 0% | 12% | 65% | 81% | |
| Philipp[30] | 2005 | 41 | PFD NOS | EPI; ADP | | | 23% | – | – | 92% |
| Koessler[66] | 2012 | 8 | PFD NOS | EPI; ADP; P2Y[g] | 50% | 38% | 50% | – | – | |
| Moenen[84] | 2017 | 16 | PFD NOS in preoperative patients | EPI; ADP | – | – | 0% | – | – | 89% |
| Moenen[84] | 2017 | 16 | PFD NOS in patients referred to coagulation clinic | EPI; ADP | – | – | 22% | – | – | 82% |
| Haas[65] | 2019 | 15 | PFD NOS | EPI; ADP | 73%[h] | 7% | 73%[e] | – | – | – |

(*Continued*)

 © 2024. Thieme. All rights reserved.

**Supplementary Table S2** (*Continued*)

| Author | Year | Patients | Disorder | Cartridges | Sensitivity | | | Specificity | | |
|---|---|---|---|---|---|---|---|---|---|---|
| | | N | | | EPI cartridge | ADP cartridge | Combined | EPI cartridge | ADP cartridge | Combined |
| Charpy[68] | 2020 | 20 | PFD NOS | EPI | 15% | | | 94 | | – |
| | | | Others | | NA | NA | NA | NA | NA | NA |
| Harrison[51] | 1999 | 3 | Noonan | EPI; ADP | 67% | 100% | 100% | 80% | 82% | – |
| Harrison[60] | 2002 | 5 | Wiskott–Aldrich syndrome | EPI; ADP | 80% | 60% | 80%[e] | 80% | 82% | – |
| Harrison[60] | 2002 | 11 | Macrothrombocytopenia NOS | EPI; ADP | 73% | 36% | 73%[e] | 80% | 82% | – |
| Harrison[60] | 2002 | 1 | May–Hegglin anomaly | EPI; ADP | 100% | 100% | 100% | 80% | 82% | – |
| Harrison[60] | 2002 | 10 | Unclassifiable platelet defect | EPI; ADP | 90% | 70% | 90%[e] | 80% | 82% | – |
| Posan[32] | 2003 | 15 | Various platelet disorders[i] | EPI; ADP | 67% | 40% | 80% | 82% | 83% | 77% |
| Dargaud[20] | 2005 | 3 | COX deficiency | EPI; ADP | 0% | 0% | 0% | – | – | – |
| Lasne[93] | 2010 | 6 | Lowe syndrome | EPI; ADP | 100% | 100% | 100% | – | – | – |
| Scavone[94] | 2014 | 3 | P2Y12 receptor defect | EPI; ADP; P2Y[c] | 67% | 67% | 100% | – | – | – |
| Perez Botero[55] | 2017 | 8 | MYH9 | EPI; ADP | – | – | 38% | – | – | – |
| Perez Botero[55] | 2017 | 1 | York platelet syndrome | EPI; ADP | – | – | 100% | – | – | – |
| Perez Botero[55] | 2017 | 1 | Quebec platelet syndrome | EPI; ADP | – | – | 100% | – | – | – |
| Perez Botero[55] | 2017 | 3 | ANKRD26 | EPI; ADP | – | – | 67% | – | – | – |
| Perez Botero[55] | 2017 | 1 | GATA-1 | EPI; ADP | – | – | 100% | – | – | – |
| Perez Botero[55] | 2017 | 1 | RUNX-1 | EPI; ADP | – | – | 100% | – | – | – |
| Kaufmann[95] | 2020 | 64 | Various platelet disorders[j] | EPI; ADP | 44% | 20% | | 76% | 86% | – |

Abbreviations: ADP, adenosine diphosphate cartridge; EPI, epinephrine cartridge; NA, not applicable; NR, not reported; VWD, von Willebrand disease; VWF, von Willebrand factor .
Note: Italics font indicates not included in weighted mean calculations.
[a]The inclusion of multiple articles authored by the same individual in this table raises concerns about the potential duplication of test results.
[b]No reference range was reported in the article; therefore, results were compared with reference range of the Radboudumc.
[c]P2Y-closure time prolonged in all.
[d]Only abnormal ADP-closure time and EPI-closure time were considered as an abnormal test result.
[e]Combined sensitivity not reported; highest sensitivity of either ADP or EPI cartridge used.
[f]Abnormal thrombocyte aggregation in light transmission aggregometry (LTA).
[g]P2Y-closure time within reference range in all.
[h]Discrepancy between sensitivity reported in outcome table (93%) and in text (73%) of the article.
[i]δ-SPD $n = 1$, ADP receptor defect $n = 1$, platelet procoagulant defect $n = 1$, GT $n = 1$, platelet adhesion signal transduction defect $n = 1$, unknown $n = 5$, Fechtner syndrome $n = 1$, Sebastian platelet syndrome $n = 1$, MHA $n = 1$, Gray platelet syndrome $n = 1$, autosomal dominant hereditary thrombocytopenia unknown $n = 1$.
[j]GT $n = 4$, GI-like defect $n = 18$, TxA2 pathway defect $n = 1$, collagen receptor defect $n = 2$, δ-SPD $n = 4$, α granule disorder $n = 5$, diminished procoagulant COAT platelets $n = 7$, complex disorder $n = 13$ (defect in number of agonists LTA / flow. Possible: GT $n = 0$, GI-like defect $n = 20$, TxA2 pathway defect $n = 13$, collagen receptor defects $n = 6$, δ-SPD $n = 1$, α granule disorder $n = 1$, diminished procoagulant COAT platelets $n = 3$, complex disorders $n = 20$.

 © 2024. Thieme. All rights reserved.
